# Mechanisms contributing to inhibition of retinal ganglion cell death by cell permeable peptain-1 under glaucomatous stress

**DOI:** 10.1038/s41420-024-02070-8

**Published:** 2024-06-28

**Authors:** Gretchen A. Johnson, Bindu Kodati, Rooban B. Nahomi, Jennifer H. Pham, Vignesh R. Krishnamoorthy, Nicole R. Phillips, Raghu R. Krishnamoorthy, Ram H. Nagaraj, Dorota L. Stankowska

**Affiliations:** 1 North Texas Eye Research Institute, School of Biomedical Sciences, University of North Texas Health Science Center, Fort Worth, TX USA; 2 Department of Microbiology, Immunology, and Genetics, School of Biomedical Sciences, University of North Texas Health Science Center, Fort Worth, TX USA; 3 Department of Pharmacology and Neuroscience, School of Biomedical Sciences, University of North Texas Health Science Center, Fort Worth, TX USA; 4 Department of Ophthalmology, School of Medicine, Anschutz Medical Campus, University of Colorado, Aurora, CO USA; 5 Department of Cell and Molecular Physiology, Loyola University, Maywood, IL USA

**Keywords:** Transcriptomics, Retina, Glaucoma

## Abstract

This study assesses the neuroprotective potential of CPP-P1, a conjugate of an anti-apoptotic peptain-1 (P1) and a cell-penetrating peptide (CPP) in in vitro, in vivo, and ex vivo glaucoma models. Primary retinal ganglion cells (RGCs) were subjected to either neurotrophic factor (NF) deprivation for 48 h or endothelin-3 (ET-3) treatment for 24 h and received either CPP-P1 or vehicle. RGC survival was analyzed using a Live/Dead assay. Axotomized human retinal explants were treated with CPP-P1 or vehicle for seven days, stained with RGC marker RBPMS, and RGC survival was analyzed. Brown Norway (BN) rats with elevated intraocular pressure (IOP) received weekly intravitreal injections of CPP-P1 or vehicle for six weeks. RGC function was evaluated using a pattern electroretinogram (PERG). RGC and axonal damage were also assessed. RGCs from ocular hypertensive rats treated with CPP-P1 or vehicle for seven days were isolated for transcriptomic analysis. RGCs subjected to 48 h of NF deprivation were used for qPCR target confirmation. NF deprivation led to a significant loss of RGCs, which was markedly reduced by CPP-P1 treatment. CPP-P1 also decreased ET-3-mediated RGC death. In ex vivo human retinal explants, CPP-P1 decreased RGC loss. IOP elevation resulted in significant RGC loss in mid-peripheral and peripheral retinas compared to that in naive rats, which was significantly reduced by CPP-P1 treatment. PERG amplitude decline in IOP-elevated rats was mitigated by CPP-P1 treatment. Following IOP elevation in BN rats, the transcriptomic analysis showed over 6,000 differentially expressed genes in the CPP-P1 group compared to the vehicle-treated group. Upregulated pathways included CREB signaling and synaptogenesis. A significant increase in *Creb1* mRNA and elevated phosphorylated Creb were observed in CPP-P1-treated RGCs. Our study showed that CPP-P1 is neuroprotective through CREB signaling enhancement in several settings that mimic glaucomatous conditions. The findings from this study are significant as they address the pressing need for the development of efficacious therapeutic strategies to maintain RGC viability and functionality associated with glaucoma.

## Introduction

Glaucoma, a complex optic neuropathy, is marked by the gradual decline of retinal ganglion cells (RGCs), deterioration of optic nerve axons, and excavation of the optic disk, culminating in visual impairment [1]. The number of patients affected by glaucoma is projected to reach 112 million by 2040 [2]. The most significant and modifiable risk factor for glaucoma is elevated intraocular pressure (IOP). However, many clinical trials and studies document neurological defects still progress in some patients despite lowering the IOP [3–5]. Therefore, there is an unmet need for novel neuroprotective treatments that protect RGCs from various types of damage. Though the exact mechanism by which neurodegeneration occurs in glaucoma is still under investigation, some potential mechanisms include neurotrophin withdrawal, nitric oxide elevation, free radical generation, oxidative stress, calcium-dependent pathways, accumulation of genetic and protein modifications, heat shock proteins deficiency, and vascular insufficiency among others [6].

Small heat shock proteins (sHSPs) play a significant role in chaperoning activity to maintain the homeostasis of cellular proteins, assist in the translocation of proteins, assemble protein complexes, and degrade proteins [7, 8]. sHSPs have a highly conserved α-crystallin domain and function as ATP-independent molecular chaperones [9]. The therapeutic potential of sHSPs comes from their ability to prevent protein aggregation and facilitate their removal through the cellular proteostasis machinery via insulin-like signaling pathways [10–12]. The chaperone activity of α-crystallins comes from the hydrophobic interaction with the aggregating proteins at specific target sequences [13, 14]. Using fluorescent probes, several hydrophobic binding sites were identified in αB-crystallin that bind to aggregating proteins [15]. Sharma’s laboratory has conducted comprehensive research on identifying peptides within α-crystallin that exhibit chaperone activity comparable to their parent proteins. They identified a core DRFSVNLDVKHFSPEELKVK sequence in αB-crystallin as a chaperone peptide [16, 17]. This peptide was later named peptain-1, or P1 [18].

In addition to the chaperone activity, alpha crystallins have cytoprotective properties because of their anti-apoptotic and anti-inflammatory properties. The elevated levels of αB-crystallin have demonstrated these cytoprotective properties in many oxidative and inflammatory studies [19–21]. Recently, cellular mechanisms contributing to neuroprotection by mini chaperones have been addressed in a few studies [22, 23]. These beneficial properties of αB-crystallin could be utilized to protect neurons vulnerable to cellular and biochemical insults in glaucoma and other neurodegenerative diseases. A study by Wu and colleagues used a full-length αB-crystallin and demonstrated the protection of RGCs from an optic nerve crush injury – one of the most severe models of traumatic injury-mediated RGC death [24]. In other studies, intravitreally injected αB-crystallin promoted optic nerve regeneration [25] and increased survival of RGCs post optic nerve axotomy [26]. Moreover, in several chronic models of glaucoma, the retinal αB-crystallin levels are decreased [26, 27]. MicroRNA studies have further confirmed the decrease of αB-crystallin at early and later stages of IOP elevation [28, 29]. This decrease could possibly be due to stress-induced changes in transcription and might be one of the causes of RGC loss. Recent studies suggest that a candidate peptide derived from a “core domain” of αB-crystallin (HspB5), named P1, has robust chaperone and anti-apoptotic activities [30–33]. In addition, P1 showed remarkable neuroprotection against glaucomatous insult to rodent RGCs in vitro and in vivo [18, 34, 35]. In previous studies, the amount of systemically delivered P1 reaching RGCs was likely low and could be further enhanced. To address this limitation, we switched to intravitreal delivery and added a cell-penetrating peptide (CPP) sequence to the amino terminus of P1 (CPP-P1). This modification aimed to improve CPP-P1’s ability to traverse the inner limiting membrane and enhance its accumulation within RGCs [36]. Our aim was to investigate the mechanism of action of CPP-P1 in primary RGC culture (in vitro), human retinal explants (ex vivo), and an ocular hypertension-induced model (in vivo) of RGC injury in rats. This provided an IOP-dependent and -independent perspective into the molecular mechanisms contributing to glaucomatous degeneration. In addition, we studied the mitigation of ocular hypertension-induced glaucomatous changes by visual function analysis, assessing axon integrity, and RNA-sequencing.

## Results

### Enhanced cell penetration and retention of CPP-P1 peptide

**Fig. 1:**
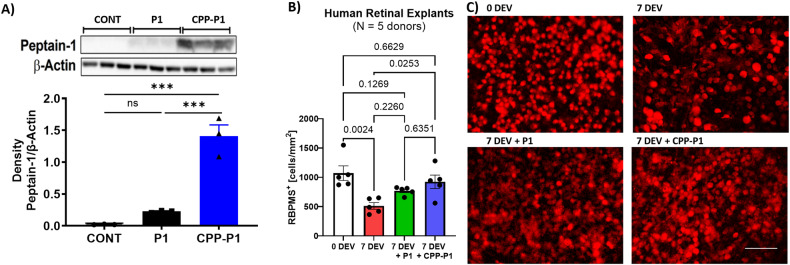
Conjugation with CPP increases the cell permeability of P1. **A** Human retinal endothelial cells were treated with vehicle (CONT), P1 or CPP-P1 (100 µM). Following incubation for 16 h, the levels of P1 in cells were detected by western blotting using an anti-P1 monoclonal antibody. Densitometric analysis was performed relative to β-actin levels (*n* = 3). Data represented as mean ± SEM, One-way ANOVA followed by Tukey’s multiple comparisons test, ***p < 0.001, ns-not significant. The entire western blot image is shown in Supplementary Figure 1. **B** Human retinal explants comparing the difference in neuroprotective efficacy of P1 with and without the CPP. **C** Representative images, RBPMS, Scale bar = 100 µm.

We previously demonstrated that P1 is RGC-permeable and accumulates within RGC somas in a time-dependent manner [18]. The current experiments were conducted to determine if the conjugation of P1 with CPP (CPP-P1) could enhance the cell permeability of P1 using a model system in which human retinal endothelial cells were incubated with the CPP-conjugated or unconjugated peptide for 16 h. There was a statistically significant difference in intracellular concentration of CPP-P1 (1.41 ± 0.17) when compared to P1 (0.23 ± 0.01, *p* = 0.0004) **(**Fig. 1 and Supplementary Figure 1). The cell permeability of CPP-P1 was approximately seven times higher than that of P1.

### CPP-P1 protects RGCs against neurotrophic factor deprivation

**Fig. 2:**
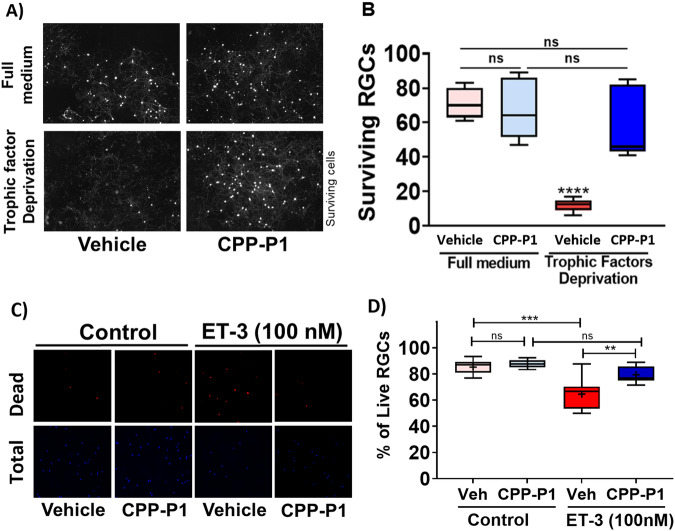
CPP-P1 protects primary rat RGCs from neurotrophic factor deprivation- and endothelin-3 (ET-3)-induced death. Primary RGCs were treated with CPP-P1 (12.5 µg/mL) or vehicle in the presence or absence of neurotrophic factors for 48 h. **A** Representative images are shown for cells in complete medium and medium deprived of neurotrophic factors treated with either vehicle or CPP-P1. **B** The bar graphs represent the number of surviving RGCs stained with CytoCalcein 450AM Viability Dye, *n* = 4 independent experiments. **C** Primary RGCs were treated with CPP-P1 (12.5 µg/mL) or vehicle in the presence or absence (control) of 100 nM ET-3 for 24 h, and representative images of cells are shown. **D** The bar graphs represent the percentage of live RGCs analyzed using the Live/Dead Assay Kit; data from a representative experiment is shown. The data represents mean ± SEM, One-way ANOVA followed by Tukey’s multiple comparisons tests, ****p < 0.0001 vs. all groups, ns- not significant. **p < 0.01, ***p < 0.001.

Following the neurite outgrowth after seven days in culture, primary retinal ganglion cells were deprived of the neurotrophic factors for 48 h either in the presence or absence of CPP-P1. In primary RGCs, neurotrophic factor deprivation led to an 83% loss of RGCs compared to cells grown in the complete medium (*p* < 0.0001). CPP-P1 treatment significantly prevented RGC loss by 64% (*p* < 0.0001) (Fig. 2A, B).

### CPP-P1 protects RGCs against ET-3 mediated death

Endothelins are vasoactive peptides known to cause neurodegeneration by both vascular and cell-mediated mechanisms [37]. To test whether CPP-P1 alleviates the effects of ET-3, we performed an analysis using primary RGCs. Primary RGCs were cultured either in the presence of a vehicle or CPP-P1 and with or without ET-3 (100 nM) for 24 h. Cell viability was then analyzed using the Live/Dead Assay Kit (Fig. 2C, D). Primary RGCs treated with ET-3 and vehicle, showed a significant 24% loss of RGCs (64.6 ± 3.2, *p* < 0.001) compared to the vehicle-treated cells without ET-3 (85.3 ± 1.7). CPP-P1 treatment significantly reduced ET-3-mediated cell death by 19% (79.5 ± 1.7, *p* < 0.01) when compared to ET-3 and vehicle-treated cells (64.6 ± 3.2). No statistical difference was observed between RGCs cultured with vehicle (85.3 ± 5.2) or CPP-P1 (87.7 ± 0.9) without ET-3.

### Effect of CPP-P1 on IOP elevation

**Fig. 3:**
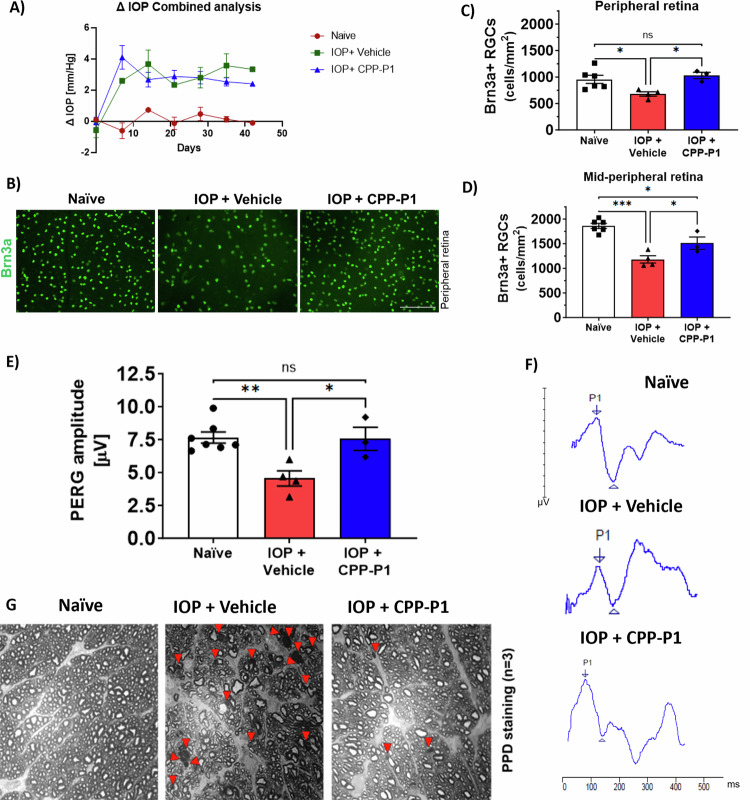
CPP-P1 enhances RGC survival and visual function and preserves axon bundles in Morrison’s model of glaucoma in rats. **A** IOP profiles of average intraocular pressure measurements in the left eye for naive rats (red) and following six weeks of IOP elevation by Morrison’s surgery either administered with CPP-P1 (blue) or vehicle (green). **B** Representative images of Brn3a staining of RGCs from rats that were injected intravitreally with either 2 μg of CPP-P1 (*n* = 3) or vehicle (*n* = 4) once a week for 6 weeks following IOP elevation. **C**, **D** Quantitation of RGCs stained with Brn3a in naive, IOP elevated vehicle administered and IOP elevated CPP-P1 administered rat eyes in peripheral and mid-peripheral eccentricities, located at two-thirds and one-third of the width of the retina from optic nerve head region. **E** Naive rats (*n* = 6) were used as control. Scale bar = 100 µm. Data represented as mean ± SEM, One-way ANOVA followed by Tukey’s multiple comparisons test, ***p < 0.001, **p < 0.01, *p < 0.05, ns-not significant, ANOVA. Rats were injected intravitreally with either 2 μg of CPP-P1 or vehicle once a week for six weeks following IOP elevation. PERG analysis was performed at six weeks following treatments; **F** Data represented as mean ± SEM, One-way ANOVA followed by Tukey’s multiple comparisons test, **p < 0.01, *p < 0.05 and ns- not significant. Naive rat eyes were used as controls. Representative PERG traces of naïve, vehicle administered, CPP-P1 administered rats. **G** Axonal degeneration accompanied by the glial scar and collapsed axons in the vehicle-administered rats compared to naive and CPP-P1 administered rats. Naive animals were used as control. Red arrowheads indicate collapsed axons, (*n* = 3).

Using the Morrison’s method, BN rats were subjected to IOP elevation in one eye. Following IOP elevation, the rats were intravitreally administered once weekly with vehicle or CPP-P1 for 6 weeks. An additional group of naive rats that did not undergo any surgical manipulation or treatment was also included in the experiment. At every studied time point, the elevation of IOP was statistically significant in the IOP-elevated and vehicle or CPP-P1 administered rats compared to naive rats. No significant difference was found in the average IOP exposures between vehicle-administered rats (714 mmHg-days, Fig. 3A) and CPP-P1-administered rats (727.5 mmHg-days), indicating no effect of the CPP-P1 administration on IOP.

### CPP-P1 attenuated RGC death in Morrison’s model of glaucoma

The extent of RGC loss following IOP elevation in rats was determined by preparing retinal flat mounts and immunostaining them with the RGC-selective marker Brn3a. Fluorescence images were taken at the peripheral (Fig. 3B) and mid-peripheral eccentricities located at a distance of two-thirds and one-third of the width of the retina from the optic nerve head, respectively. At the peripheral eccentricity, naive animals exhibited an average RGC count of 955.4 ± 188 cells/mm^2^. However, rats with elevated IOP following Morrison’s surgery and treated with a vehicle for six weeks demonstrated a significant reduction in RGC counts, with a decrease of 29% to 680 ± 81 cells/mm^2^ (*p* < 0.05) (Fig. 3C). By contrast, the IOP-elevated and CPP-P1-treated rats showed an average RGC count of 1028 ± 104 cells/mm^2^, indicating significant protection of 34% (*p* = 0.03) against RGC loss compared with the IOP-elevated vehicle-treated rats (Fig. 3C).

In the mid-peripheral eccentricity, naive animals had an average RGC count of 1861 ± 127 cells/mm^2^, while IOP-elevated and vehicle-treated rats showed a significantly lower RGC count of 1180 ± 150 cells/mm^2^ (*p* < 0.001) (Fig. 3D). This RGC loss was significantly mitigated in rats with IOP following treatment with CPP-P1. Specifically, the RGC counts in the CPP-P1 treated group was 1511 ± 218 cells/mm^2^ (Fig. 3D), which represents a statistically significant protection (*p* = 0.046) when compared to the average RGC count in rats with elevated IOP that received only the vehicle treatment. IOP-elevated vehicle-injected animals showed a decline in RGC counts by 37% (*p* < 0.001) compared to naive rat eyes. CPP-P1-treatment in IOP-elevated eyes significantly protected against RGC loss by 22% (*p* < 0.05) compared to IOP-elevated vehicle-treated rats.

### CPP-P1 alleviated the RGC function decline in IOP-elevated rats

RGC function was assessed by performing PERG in rats treated with vehicle or CPP-P1 during IOP elevation. After 6 weeks of IOP elevation, a significant (*p* = 0.01) 41% decline was observed in the peak amplitude in IOP-elevated and vehicle-treated rats compared to naive rats. By contrast, the RGC function of IOP-elevated and CPP-P1-treated rats showed significant (*p* = 0.04) preservation in the peak amplitude compared to IOP-elevated and vehicle-treated rats (IOP-elevated vehicle-treated: 4.57 ± 0.8 μV; IOP-elevated CPP-P1-treated: 7.55 ± 0.8 μV; naive 7.65 ± 0.4 μV; Fig. 3E, F), indicating the neuroprotective effects of CPP-P1 against a decline in RGC function. No significant difference in amplitude was observed when comparing naive animals to those with elevated IOP treated with CPP-P1 (*p* = 0.98). However, we did not find any difference in the latencies between the groups (IOP-elevated vehicle-treated: 56.17 ± 3.5 ms; IOP-elevated CPP-P1-treated: 42.5 ± 9.4 ms; naive: 51.9 ± 1.4 ms).

### CPP-P1 protects against degeneration of the optic nerve following IOP elevation in rats

After six weeks of elevated IOP, rats were euthanized to isolate optic nerves. The optic nerve sections were stained with PPD and analyzed. The results showed that compared to the naive animals, the IOP-elevated rats treated with vehicle had higher numbers of disrupted optic nerve axonal bundles, intense myelin staining, and glial scar formation (Fig. 3G). However, the IOP-elevated rats treated with CPP-P1 showed less prominent glial scarring than the vehicle-treated rats. Additionally, the rats treated with CPP-P1 showed fewer collapsed axons (indicated by red arrowheads) than those treated with the vehicle. These results suggest that the intravitreal administration of CPP-P1 promotes axoprotection during IOP elevation, which was reflected by the decrease in collapsed axons.

### CPP-P1 enhances RGC survival in human retinal explants

**Fig. 4:**
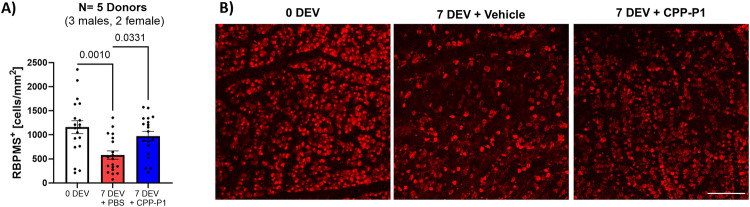
CPP-P1 is neuroprotective in human retinal explants. Human retinal explants were cultured for 7 days in a medium containing either vehicle or CPP-P1. **A.** RGCs were quantified (0 DEV) (DEV = days ex vivo) or after 7 days culture (7 DEV) in the presence of vehicle or CPP-P1 (*n* = 15-20, 5 human donors). **B** Representative images of RGCs (RBPMS; red), scale bar = 250 μm.

In addition to the animal models, we explored the efficacy of CPP-P1 on RGC survival within human retinal explants, further exploring its therapeutic and translation potential. Following 7 days of treatment with vehicle or CPP-P1, human retinal explants were fixed in 4% PFA overnight. The retinal explants were then immunostained for RBPMS, an RGC marker, and images were captured in a confocal microscope. At 0 days ex vivo (0 DEV), the control retinas had an average cell count of 1113 ± 105 cells/mm^2^ (Fig. 4A). After 7 DEV, the CPP-P1-treatment significantly prevented RGC loss by 34% (*p* = 0.03, 943 ± 80 cells/mm^2^), compared to the vehicle-treated eyes at 50% loss with only 561 ± 71 cells/mm^2^ (Fig. 4B).

### CPP-P1 treatment altered gene expression in RGCs

**Fig. 5:**
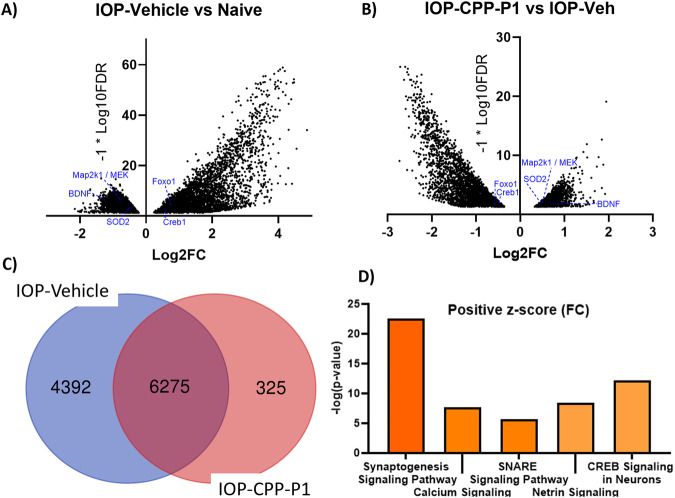
Volcano plots of Differentially Expressed Genes (DEGs) with FDR < 0.05 and top 5 IPA pathways. **A** Significant DEGs were expressed in the IOP-Vehicle group as compared to the naive, each gene represented by a single dot on the graph; positive fold change (x-axis) indicates increased expression, and negative indicates decreased expression. **B** Significant DEGs in the IOP-CPP-P1 group, representative genes shown. **C** Venn diagram of the plotted DEGs showing 4392 unique to the IOP-Vehicle group, 6275 shared, and 325 unique to the IOP-CPP-P1 group. **D** Qiagen**’**s IPA-generated pathways are colored orange to signify the upregulation or increased expression. The two pathways with the highest p-values, synaptogenesis, has a *p* = 0.3E-22, and CREB signaling in neurons has a *p* = 0.6E-12 level of significance.

We proceed to the gene expression analysis to elucidate the impact of CPP-P1 on cellular transcriptional activity. An RNA-seq analysis was conducted on adult rat RGCs isolated after one week of IOP-elevation. The results showed that almost 11,000 genes were altered in comparison to the naive group (Fig. 5A), and 6,600 genes were altered by CPP-P1 from the IOP-elevated, vehicle-treated group (Fig. 5B). Of these differentially expressed genes (DEGs), 6275 genes that were altered due to IOP-elevation were also changed by CPP-P1 (Fig. 5C). This could imply that some pathological processes were possibly reversed, as the expression of what was altered (either increased or decreased) in the vehicle group (compared to the healthy naive group) demonstrated a change in the CPP-P1-treated group.

### CPP-P1 promotes cell survival signaling

**Fig. 6:**
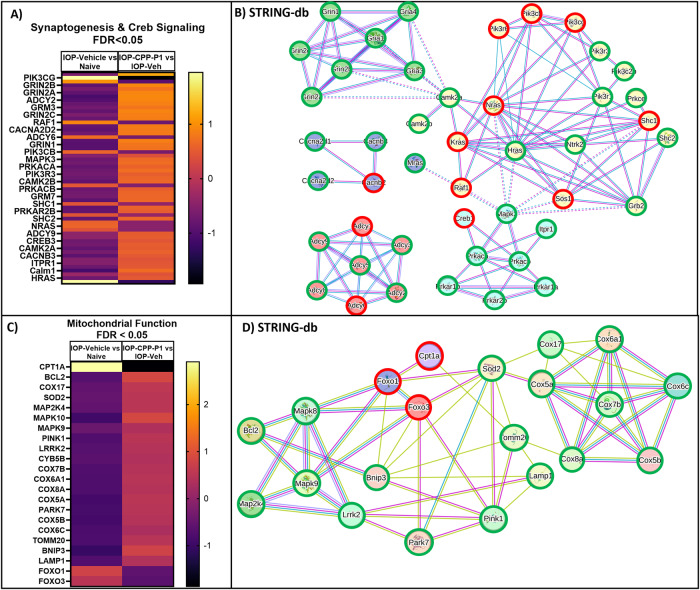
Genes related to Creb signaling and Synaptogenesis as indicated by IPA and genes related to mitochondrial function indicated by MitoMiner; padj/FDR < 0.05. **A** A heatmap analysis demonstrating DEG in IOP and vehicle-treated group in comparison to IOP and CPP-P1 treated rats. Fifty-seven genes assigned to synaptogenesis and CREB signaling are shown here. **B** STRING-db generated a network of the genes in the CREB pathway. Pink lines indicate experimentally determined connections and blue lines indicate known connections from curated databases. Yellow clusters are associated with MAPK signaling and GPCRs, red clusters are associated with PKA activation, green clusters are associated with glutamate receptor signaling, blue clusters are related to high voltage-gated calcium channel activity, and teal clusters are associated directly with Creb1 phosphorylation. **C** A heatmap analysis demonstrating differential gene expression in IOP and vehicle-treated group in comparison to IOP and CPP-P1 treated rats. **D** STRING-db generated network of the genes in the mitochondrial function category altered by CPP-P1 treatment. Pink lines indicate experimentally determined connections, blue lines indicate known connections from curated databases, and yellow lines indicate genes often mentioned together in publications. STRING-db gene outlines indicate a change of expression; green represents upregulation, and red represents downregulation.

Some pathways identified by Ingenuity Pathway Analysis (IPA) were further investigated and confirmed. Significantly upregulated pathways following CPP-P1 treatment include synaptogenesis, calcium signaling, netrin signaling, SNARE signaling, and CREB signaling in neurons (Fig. 5D). Compared to the naive group, the IOP and vehicle-treated group demonstrated a decreased expression of several members of the CREB signaling pathway (Creb-1, c-RAF, MEK1/2, ERK1/2, and p90RSK). This decline was prevented by CPP-P1 treatment and was visualized by contrasting expressions in the heatmap (Fig. 6A, light yellow increased, dark purple decreased). As indicated by Search Tool for the Retrieval of Interacting Genes/Proteins - database (STRING-db) (Fig. 6B), top Gene Ontology (GO) functions included glutamate receptor activity, calcium channel activity, small molecule binding, adenylate cyclase activity, protein binding, and enzyme binding. The top KEGG pathways suggested included phospholipase D signaling, cAMP signaling, estrogen signaling, glutamatergic synapse, and growth hormone synthesis, secretion, and action. KEGG pathways also highlighted (but not listed as high) MAPK signaling and long-term potentiation, both of which are pathways indicated as being active following phospholipase D, cAMP, or growth hormone signaling.

Qiagen’s IPA indicated that many of the 11,000 significant DEGs from RNA-Seq were related to mitochondrial function. In Fig. 6C the expression of select members of mitochondrial function (e.g., *COXs, PINK1, TOMM20, LAMP1, FOXO1*, etc.) in the IOP-elevated and vehicle-treated group is shown on the left, with most members being decreased, and the IOP-elevated and CPP-P1 group on the right, with most members being increased. In Fig. 6D, the same genes were plugged into STRING-db software to demonstrate functional and/or physical connections between the proteins to visualize how they may interact to influence mitochondrial function.

### CPP-P1 increases *Creb1* and *Foxo1* RNA expression in rat and human retinas

**Fig. 7:**
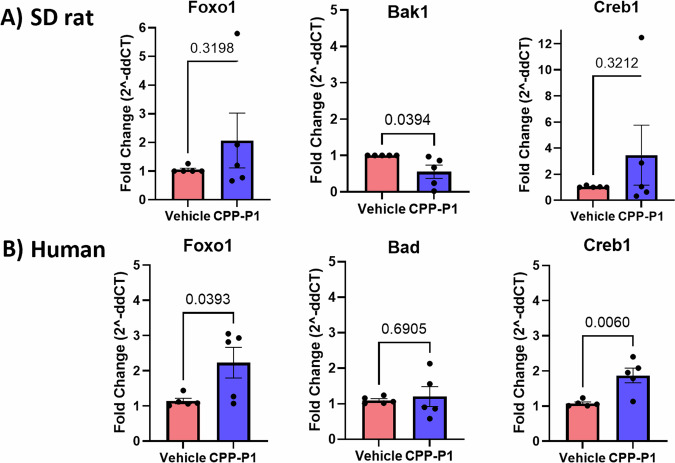
Confirmation of Creb1, BCL-2 pro-apoptotic genes, and Foxo1 RNA expression. **A** qPCR of RNA isolated from SD rat primary RGCs showed elevated *Foxo1* and *Creb1* expression in the IOP and CPP-P1 treated groups compared to the vehicle-treated groups with decreased *Bak1* expression. **B** qPCR of human retinal explant RNA showed elevated *Foxo1* and *Creb1* expression in the IOP and CPP-P1 treated groups compared to the vehicle-treated groups with no difference in *Bad* expression. Cyclophilin A (PPIA) was used as the reference gene in humans, and GAPDH was used in rats to calculate delta CT.

Quantitative PCR further confirmed the RNA-seq findings of the increased expression of *Creb1* in IOP-elevated and CPP-P1-treated rats compared to that of the IOP-elevated and vehicle-treated group (Fig. 7). In the NF deprivation model, in primary RGCs treated with CPP-P1, compared to vehicle-treated groups, mRNA expression of *Creb1* increased (3.5-fold, *p* = 0.32), *Foxo1* increased (2.1-fold, *p* = 0.32), and *Bak1* showed a significant decrease (0.55-fold, *p* = 0.04). In the human retinal explants treated with CPP-P1, *Creb1* expression was increased (1.87-fold, *p* = 0.006), *Foxo1* expression was increased (2.22-fold, *p* = 0.04), but *Bad* expression was not altered (1.21-fold, *p* = 0.69). These results suggest that CPP-P1 caused significant changes in gene expression through the involvement of other cell types in mediating the neuroprotective effects.

### CPP-P1 increases MEK/ERK/CREB signaling in primary RGCs

**Fig. 8:**
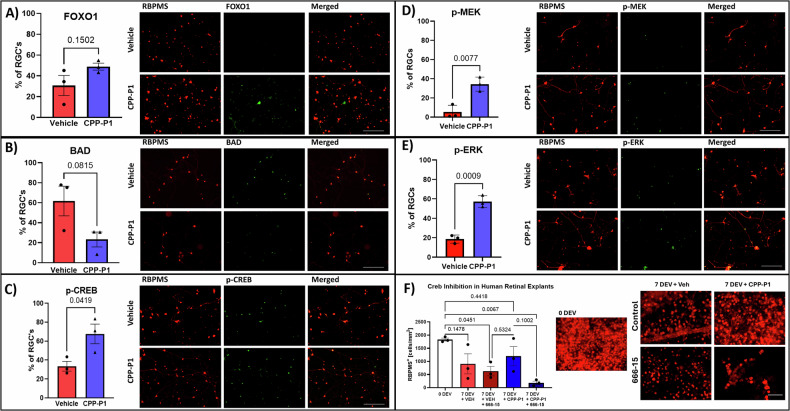
Confirmation of p-MEK/p-ERK/p-CREB, FOXO1, and BAD expression in protein. RGCs were treated with either CPP-P1 or vehicle and underwent 48 hours of neurotrophic factor (NF) deprivation. Only cells positive for RBPMS were considered, and the percentage of those cells expressing each target are shown in the plots. A-C has 3 biological replicates while D and E have 1 biological replicate consisting of 4 technical replicates. **A** analysis showed an elevated Foxo1 expression in the -NF and CPP-P1-treated groups compared to the -NF and vehicle-treated groups. **B** analysis showed a decreased BAD expression in the -NF and CPP-P1-treated groups compared to the -NF and vehicle-treated groups. **C** analysis showed an elevated p-CREB expression in the -NF and CPP-P1-treated groups compared to the -NF and vehicle-treated groups. **D** analysis showed an elevated p-MEK expression in the -NF and CPP-P1-treated groups compared to the -NF and vehicle-treated groups. **E** analysis showed an elevated p-ERK expression in the -NF and CPP-P1-treated groups compared to the -NF and vehicle-treated groups. Representative images of immunostaining taken at 20X magnification, scale bar = 200 μm. **F** Inhibition of Creb via 666-15 in human retinal explant punches showed that the protective effects of CPP-P1 are diminished in the presence of 666-15, scale bar = 100 µm.

Immunocytochemical analyses were used to assess protein expression (*n* = 3 biological replicates) for the active phosphorylated form of Creb (p-CREB), p-MEK, p-ERK, FOXO1, and the pro-apoptotic BAD protein **(**Fig. 8). The expressions of p-MEK, p-ERK, p-CREB, and FOXO1 were increased by 555% (*p* = 0.0077), 174% (*p* = 0.0009), 102% (*p* = 0.04), and 59% (*p* = 0.15), respectively, and BAD expression was decreased by 62% (*p* = 0.08) in CPP-P1-treated RGCs.

### Inhibition of CREB in human retinal explants using 666-15

Following 7 days of treatment with vehicle or CPP-P1 and with or without CREB inhibitor 666-15 [250 nM], human retinal explants were fixed in 4% PFA overnight. The retinal explants were then immunostained for RBPMS, an RGC marker, and images were captured in a confocal microscope. At 0 days ex vivo (0 DEV), the control retinas had cell counts of 1830 ± 56 cells/mm^2^, while the vehicle-treated groups without and with CREB inhibitor 666-15 had counts of 908 ± 382 cells/mm^2^ (50% loss, *p* = 0.15) and 630 ± 179 cells/mm^2^ (66% loss, *p* = 0.05), respectively. CPP-P1-treated groups without and with 666-15 had counts of 1199 ± 365 cells/mm^2^ (34% loss, *p* = 0.38) and 185 ± 65 cells/mm2 (90% loss, *p* = 0.007), respectively, indicating a significant attenuation of CPP-P1’s effects in the presence of the CREB inhibitor. There was considerable variability in this experiment, likely due to the low number of replicates and a high concentration of 666-15, as indicated by the dramatic decrease in cells compared to the vehicle-treated alone. Additionally, there may have been a direct interaction/binding between CPP-P1 and 666-15, attenuating CPP-P1 action. For future experiments, one option is to pre-treat with a lower inhibitor dose, followed by CPP-P1 treatment.

## Discussion

The mainstay for glaucoma treatment has been the reduction of IOP using topical eye drops or surgical interventions. Lowering IOP, however, only delays the progression of RGC death and visual function loss, and neurodegenerative effects persist in a subset of patients. There is a pressing need for additional treatment modalities to complement existing IOP-lowering therapies in glaucoma. While individual neuroprotective agents could be efficacious, a combination of therapeutic agents may ultimately be required to target the multiple pathways leading to RGC death. Our previous studies have shown that P1 has a neuroprotective effect when administered through intraperitoneal or intravitreal injections in rodent models of glaucoma [18, 35, 38]. In this study, we aimed to enhance the neuroprotective effects of peptain-1 by increasing its cell permeability. We achieved this by attaching a cell-penetrating peptide (CPP) to it.

CPPs are short sequences of 5 to 30 amino acids containing multiple arginine or lysine residues and are non-selectively taken up by cells directly or through an endosomal mechanism [39, 40]. The attachment of CPP (VPTLK) to mini-αA-peptide increased its cellular uptake and decreased H_2_O_2_-induced apoptosis [36, 41]. We reasoned that adding CPP to P1 would increase its permeability through the inner limiting membrane based on the approximately seven-fold increase in its cell permeability in retinal endothelial cells (Fig. 1A); CPP-P1 would likely provide superior results and consequently enhance therapeutic benefits. CPP-P1 was more efficacious than P1 alone in protecting human RGCs from neurodegeneration in the organotypic explant model (Fig. 1B). However, we acknowledge that a comparative study in the glaucoma animal model would be required to better understand the benefit of CPP-P1 over P1.

In glaucoma, IOP-dependent and IOP-independent factors contribute to the pathological changes in the retina. A recent study using Müller glial cells showed an increase in the neurotrophic factor, CNTF, upon treatment with αB-crystallin [42, 43]. The sequencing data generated in this study showed a trend in increase in CNTF mRNA with a significant increase in mRNA for CNTF receptor – possibly priming RGCs for support from exogenous CNTF produced by Müller glial cells. Neurotrophic factor BDNF was also significantly increased in the sequencing data. Whether CPP-P1 enhances BDNF, CNTF, and/or its receptor to protect RGCs will need to be investigated in a future study.

In both acute and chronic models of glaucoma, loss of RGCs leads to functional visual field loss, influenced by axonal degeneration. Our previous experiments demonstrated that P1 inhibits glial activation and mitochondrial dysfunction [18, 35]. Axonal transportation deficits have been shown in various animal models of glaucoma and we demonstrated the amelioration of these deficits by administration of P1 [18, 35]. It is unclear if the beneficial effects of CPP-P1 are also observed in other paradigms of ocular neurodegeneration.

Another factor contributing to retinal neurodegeneration is oxidative stress caused by increased ROS [44]. P1 showed protective effects against oxidative stress caused by tertiary butyl hydrogen peroxide or H_2_O_2_ treatment in retinal pigmented epithelial cells [45, 46]. In the ischemic/reperfusion model, the intravitreal injection of αB-crystallin protected RGCs from the ischemic effect and thinning of the retinal layers [47]. αB-crystallin has also been observed to act within mitochondria to mitigate oxidative stress [30]. Whether CPP-P1 would similarly reduce the deleterious effects of ROS in animal models needs to be verified.

Endothelins are known to induce oxidative stress and the production of reactive oxygen species, contributing significantly to the development of vascular dysfunction and cardiovascular disease [48]. In this study, we focused on the survival of the RGCs under oxidative stress by treatment with endothelin (ET-3). CPP-P1 protected primary RGCs from endothelin-mediated neurodegeneration and improved cell survival by 19%.

In our previous study, we demonstrated the protective effects of P1 on mitochondria by restoring the expression levels of COX 6b2 in an ocular hypertensive rodent model [18]. Genes identified from RNA-sequencing that were increased with CPP-P1 treatment in relation to mitochondrial function included anti-apoptotic *Bcl2, SOD2, PINK1*, and *TOMM20*. One target (Foxo1) was chosen for preliminary protein confirmation due to its known role in ROS scavenging. The expression of *Foxo1* and *Foxo3* is also of interest because FOXOs are required for clearance of ROS, and Foxo3 specifically has been observed to cause axonal degeneration in the absence of neurotrophic factors [49]. Both *Foxo1* and *Foxo3* levels were decreased in sequencing data (7 days injury/treatment) but when *Foxo1* levels were analyzed after 48 h of CPP-P1 treatment in primary RGCs, both RNA and protein expression were increased. To explain this observation, there could be an early activation of FOXO1 due to oxidative phosphorylation and subsequent inhibition of FOXO1 by PI3K-Akt or MAPK signaling.

Sequencing data and pathway analysis also supported several significant positive alterations in gene expressions consistent with CREB signaling in neurons, synaptogenesis, SNARE signaling, Netrin signaling, and calcium signaling. Our findings of downstream signaling pathways activated by CPP-P1 have significant implications, given the crucial role of the CREB family in synaptic plasticity, memory, and learning [50, 51]. A recent study demonstrated that CaMKII-CREB signaling is attenuated after excitotoxic injury [52] and IOP elevation in rats [53]. Studies have also been investigating the connection between CREB signaling and neurotrophins [54], with publications as early as 2000 proposing CREB signaling as key for neuronal survival [55]. Our findings of MEK, ERK, and CREB activation by CPP-P1 provide a promising indication to continue pursuing the therapeutic potential of neuroprotection in RGCs (Supplementary Figure 2).

Excess and dysregulated calcium homeostasis have been observed in various neurodegenerative contexts [56]. In the IOP and CPP-P1 treated rats, calcium signaling was significantly increased in the RNA-sequencing data interpretation by IPA compared to the IOP and vehicle-treated group, with predicted increases in ryanodine receptors (Ryr), which release calcium from the endoplasmic reticulum, transient receptor potential channels (Trp) which are calcium-permeable, and sodium-calcium exchangers (NCX). This parameter is of interest due to the increase in calcium signaling in the IOP- and CPP-P1 group compared to the IOP- and vehicle group. Dysregulated calcium signaling is a known contributor to cell death [57, 58]. It is possible that CPP-P1 upregulated NCX and the voltage-gated calcium channel expression to restore calcium homeostasis and prevent calcium overload-mediated damage to RGCs [57].

Based on the ability of αB-crystallin to reduce cellular stress and inhibit apoptosis, we expected a notable decrease of pro-apoptotic proteins upon treatment with CPP-P1 [41, 59]. This study showed a prominent reduction in BAD protein expression after 48 h in rat primary RGCs, with slight alteration at the RNA level in human retinas and a significant decline in Bak1 mRNA in primary RGCs. Our study reveals cellular, molecular and signaling mechanisms underlying the neuroprotective effects of CPP-P1 under conditions similar to those encountered in glaucoma.

## Conclusions

In this study, we investigated the neuroprotective properties of peptain-1 conjugated with a cell-penetrating peptide (CPP) in various models of retinal ganglion cell (RGC) neurodegeneration. Our findings indicate that administering CPP-P1 attenuates RGC loss, preserves function, and reduces axonal degeneration in ocular hypertensive rats. Additionally, CPP-P1 improves RGC survival in human retinal explants. We observed that CPP-P1 exhibits enhanced penetration in human ocular cells and provides protective effects in primary RGCs under neurotrophin-deprived and ET-3-induced oxidative stress conditions. Our study demonstrated that CPP-P1 promotes cell survival by decreasing pro-apoptotic protein expression and enhancing CREB signaling. Thus, CPP-P1 holds potential for further development as a neuroprotective agent in glaucoma and other neurodegenerative diseases of the central nervous system.

## Materials and Methods

### Animals

All animal experiments were carried out in accordance with the policies of the Association for Research in Vision and Ophthalmology (ARVO) resolution on the use of animals in ophthalmic and vision research and approved by the Institutional Animal Care and Use Committee (IACUC) at the University of North Texas Health Science Center. Brown Norway rats were purchased from Charles River (Wilmington, MA) or Envigo (Indianapolis, IN). The animals were housed in rooms with controlled temperature, humidity, and constant dim light. Food and water were provided ad libitum.

### Peptide

The P1 (DRFSVNLDVKHFSPEELKVK) and CPP-P1 (VPTLK-DRFSVNLDVKHFSPEELKVK) peptides were obtained from Peptide 2.0, Inc. (Chantilly, VA) at 95-98% purity and reconstituted in a vehicle (PBS) and kept at a stock concentration of 1 µg/µL at −20 °C.

### In vitro studies

#### Cell Penetration and Retention of CPP-P1

Human retinal endothelial cells were incubated with vehicle, P1, or CPP-P1 (100 µM) in serum-free DMEM/F12 (low glucose) medium for 16 h. Cells were treated with 1X RIPA buffer containing protease inhibitor cocktail and cell lysates were prepared. Western blot was performed using an anti-P1 monoclonal antibody [32] with β-actin as the loading control (*n* = 3).

#### Isolation of primary retinal ganglion cells

Primary RGCs were isolated from Sprague-Dawley rat pups, postnatal 5-7 days, by a modified two-step immunopanning method adapted from Barres et al. 1998 [18, 60–62].

#### Neurotrophic factor deprivation model in primary RGCs

Primary RGCs were cultured either in complete media containing neurotrophic factors (NF) or media deprived of NF for 48 h in the presence of either CPP-P1 (12.5 μg/mL) or vehicle. Following incubation, the culture media was removed, and RGCs were washed with serum-free DMEM medium. RGC survival was assessed using CytoCalcein 450AM Viability Dye Kit (65-0854-39, Invitrogen, CA) according to the manufacturer’s instructions and imaged using Cytation 5 cell imaging multi-mode reader (Agilent Technologies).

#### Oxidative stress model in primary RGCs following endothelin-3 (ET-3) treatment

Following 7 days in culture to facilitate neurite outgrowth, primary RGCs were treated with either DMEM serum-free media alone or with 100 nM ET-3 (#4013291.0500, Bachem, Torrance, CA) and incubated at 37 °C for 24 h in the presence of either CPP-P1 (12.5 µg/mL) or vehicle. Following incubation, cells were washed twice using Dulbecco’s Phosphate Buffered Saline (DPBS) (#14278072, Gibco, NY) and incubated with a Live/Dead assay Kit (L3224, Thermo Fisher Scientific, Waltham, MA) for 30 min. Living cells were treated with a mixture of Calcein-AM, which labels living cells with green fluorescence, and ethidium homodimer (EthD-1), which labels dead/dying cells with red fluorescence. Cell nuclei were labeled with a Hoechst stain to observe and obtain total cell numbers. Images were taken using a fluorescent microscope, and cell counts were performed using ImageJ/Fiji (National Institutes of Health, Bethesda, MD) [63].

#### Quantitative PCR (qPCR)

RNA isolation was carried out using the TRIzol reagent from treated (vehicle or CPP-P1, 12.5 µg/mL) primary RGCs isolated from Sprague-Dawley pups as well as from 3 mm human retinal punches treated for 48 h with either vehicle or CPP-P1, 12.5 µg/mL. Next, cDNA synthesis and PrimePCR assays were done with SsoAdvanced Universal SYBR Green Supermix (Bio Rad) for detection with Bio Rad’s CFX90 Real-Time System C1000 Touch Thermal Cycler. The targets for confirmation of changes in gene expression were chosen based on the RNA-sequencing (described later in the text) expression (e.g., creb1, bad, foxo1, etc.). Targets chosen include: Creb1, Foxo1, and Bak1 (in rats) /Bad (in humans) and housekeeping genes GAPDH (in rats) and Cyclophilin A (PPIA, in humans) (Bio Rad PrimePCR SYBR Green Assay).

#### Immunocytochemistry

RGCs grown on glass coverslips were fixed in 4% paraformaldehyde at room temperature for 20 min, washed with PBS three times, permeabilized with 0.1% sodium citrate, 0.1% Triton X-100 in 1X PBS for 5 min, blocked with blocking buffer (5% normal donkey serum, 0.1% Triton X-100 in 1X PBS) for 1.5 h, and incubated with one of the following primary antibodies: RBPMS (1:200 dilution), p-MEK, p-ERK, p-CREB, FOXO1, or BAD (Cell Signaling Technologies, 1:250) at 4 °C overnight. The corresponding species-specific fluorescently labeled secondary antibodies (Alexa-488, −546, and −647, 1:1000; Invitrogen, Carlsbad, CA, USA) were subsequently added, incubated for 2 h at room temperature in the dark, and mounted with FluoroSave reagent (Sigma-Aldrich Corp., St. Louis, MO, USA) on glass slides. Images were taken by Cytation 5 at 20X magnification and analyzed in ImageJ.

### In vivo studies

#### Morrison’s model of ocular hypertension in rats

Morrison’s model of IOP elevation was used to induce ocular hypertension in Brown Norway rats (5-7 months old) [64, 65]. Briefly, the rats were anesthetized with 4% isoflurane for induction and maintained at 2.5% isoflurane with an oxygen level of 0.8-1 L/min. Following the incision on the conjunctiva, a small episcleral vein was targeted to inject 50 μL of 1.8 M saline using a glass needle (TIP01TW1F, WPI) at a rate of 309 μL/min for 10–20 S. To reduce diurnal fluctuations in aqueous outflow facility, the rats were housed with constant dim light (90 lux) throughout the experiment.

#### Intraocular Pressure (IOP) Measurements

IOP in rats was measured twice weekly with a TonoLab tonometer (Icare Finland Oy, Espoo, Finland) under isoflurane anesthesia. Each IOP measurement from the device is presented as the mean of six individual measurements, and ten IOP measurements were taken for each eye. The cumulative IOP was calculated by taking the total IOP exposure over the period of time (days) for each eye. The extent of elevation was calculated based on the difference between the IOP-elevated eye and contralateral control eye (extent of IOP exposure/days of IOP = AUC of the IOP-elevated eye - AUC of the control eye) and expressed in mmHg-days.

#### Intravitreal Injections of CPP-P1 or vehicle

Brown Norway rats were anesthetized using isoflurane, and IOP was elevated in one eye by the Morrison method (injection of hypertonic saline through episcleral veins). One week following IOP elevation, the IOP-elevated eye was injected with either CPP-P1 (2 μg/4 μL eye) or vehicle (4 μL) using a Hamilton syringe. Briefly, a single drop of 0.5% proparacaine hydrochloride (Alcon Laboratories, Inc., Fort Worth, TX, USA) and 1% tropicamide was applied to both eyes. A 33-gauge needle connected to a 10 μL Hamilton syringe was used to intravitreally inject 4 μL of either CPP-P1 or vehicle into the vitreous through the sclera, 1 mm behind the limbus region. The injection was carried out slowly to avoid touching the lens during the needle insertion. After the injection, the needle was held for 1 min and gradually withdrawn from the vitreal chamber. After the injection, a triple antibiotic (bacitracin zinc, neomycin sulfate, and polymyxin B sulfate) was applied at the injection site, and the animals were allowed to recover from the anesthesia. Six weeks following the intravitreal injection, animals were euthanized by an overdose of 120 mg/kg body weight of Fatal-Plus (pentobarbital, Covetrus, Dublin, OH) administered intraperitoneally followed by an intracardial injection.

#### Pattern electroretinography

Pattern electroretinography (PERG) was performed following IOP elevation to analyze the function of RGCs by measuring amplitude and latency as described previously in our lab [64]. Briefly, rats were anesthetized by intraperitoneal injection (100 μL/100 g body wt) of a ketamine/xylazine cocktail (VEDCO, Saint Joseph, MO) with final concentrations of 55.6 mg/mL and 5.6 mg/mL, respectively. Rats were placed on an adaptive heated stage. PERG measurements were conducted using the Jörvec instrument (Jörvec, Miami, FL) per the manufacturer’s instructions.

#### Retinal flat mount immunostaining

Six weeks post-IOP injections, BN rats (*n* = 3-7 eyes per treatment group) were euthanized, eyes were enucleated and small incisions were made using the 22 G needle near the limbus region and immediately fixed overnight in 4% paraformaldehyde at 4 °C. After three washes with 1x PBS following the overnight incubation, retinas were carefully separated from the globe and permeabilized (0.1% sodium citrate, 0.1% Triton X-100 in PBS for 5 min) at room temperature and incubated overnight in blocking buffer (5% normal donkey serum and 5% BSA in PBS) at 4 °C. The retinas were then incubated with the primary antibody, goat anti-Brn3a (1:200, SC-31984, Santa Cruz Biotechnology, Inc. TX), for three days at 4 °C. After three washes with PBS, the retinas were incubated overnight with one of the secondary antibodies: Alexa 488 conjugated donkey anti-goat antibody (1:1000 dilution, A11055, Invitrogen) at 4 °C. After washes, cuts were made in the four quadrants (superior, inferior, nasal, and temporal) and the retinal flat mounts were mounted using Prolong Gold anti-fade (Thermo Fisher Scientific).

#### Quantification of RGCs in retinal flat mounts

Images of flat mounts were captured using magnification × 20 in a Cytation 5 microscope. Images were taken at two different eccentricities, at one-third (mid-peripheral) and two-thirds (peripheral) of the distance between the optic nerve head and the periphery of the retina. Two images were captured at each eccentricity, in each of the four quadrants. RGC counts were determined by manual counting by a masked observer.

#### PPD staining of the optic nerve for the assessment of axonal damage

Our previous studies described axonal degeneration using PPD staining, which stains the myelin around the axon [18, 64]. Briefly, Brown Norway rats injected with vehicle and CPP-P1 (2 μg/eye) were maintained for six weeks. The rats were then euthanized, and their eyes were enucleated, after which the optic nerves were excised from the posterior side of the globe. The optic nerves were immediately fixed in 2% paraformaldehyde and 2.5% glutaraldehyde in 0.1 M sodium cacodylate buffer. Before dehydration, the optic nerves were transferred to 2% osmium tetroxide in PBS for one hour and embedded in Epon. Optic nerve cross-sections were obtained using an ultramicrotome and stained with 1% PPD. Images of the stained sections were taken in a Zeiss LSM 510 META confocal microscope using an oil immersion magnification × 100.

#### Primary RGC isolation from adult rats

Primary RGCs were isolated as described previously, with some modifications. Retinas were isolated from Brown Norway rats (*n* = 9, 5-7 months) subjected to various treatments, including Naive (healthy control), IOP-elevated vehicle-treated, and IOP-elevated and CPP-P1-treated groups. RGCs were captured by the previously described immunostaining technique and treated with TRIzol reagent (Thermo Fisher Scientific) for RNA collection and isolation.

#### RNA Isolation

After the addition of TRIzol, the PureLink™ RNA Mini Kit (Thermo Fisher Scientific) was used and the protocol to precipitate, wash, and isolate RNA was followed as described by the manufacturer. The RNA samples were immediately stored at −80 °C.

#### Tape station

The buffer and screen tape were first equilibrated to room temperature for 30 minutes before use. Then, 5 µL of buffer was added to microtubes, and 1 µL of sample or ladder was added. The samples and ladder were then vortexed for one minute and spun down before being heated to 72 °C for three minutes, followed by two min of storage on ice. Samples were then inserted into the instrument for injection into the screen tape and RNA quality analysis by assigning an RNA integrity number (RIN). For RNA sequencing, RIN values greater than seven were desired (Naive group values were 8.3, 9.3, and 9.4; IOP-elevated and vehicle-treated group values were 5.2, 4.1, and 5.0; the IOP-elevated and CPP-P1-treated group values were 7.7, 7.9, and 7.9). While RIN scores of less than 7 are not ideal, normalizing methods built into DESeq2 account for differences in sequencing depth and RNA composition, which should minimize the potential confounding effects [66].

#### RNA-sequencing

RNA sequencing was carried out at Texas A&M University’s Molecular Genomics Core Facility using the Illumina Stranded Total RNA Prep, Ligation with Ribo-Zero™ Plus. No spike-in controls were used, and the library quality and quantity were assessed with an Agilent bio-analyzer. Samples were pooled at equimolar ratios for subsequent sequencing on an Illumina NextSeq 550 high-output flow cell, generating approximately 50 million sequencing reads per sample.

#### Bioinformatics

Illumina sequencing data in a FASTQ format was analyzed in Galaxy (usegalaxy.org). The subsequent steps were done using programs available on the Galaxy interface. Quality control check (QC) was performed using the FastQC algorithm. Following QC checks of the reads, adapters were trimmed, and results were mapped or aligned to a reference genome (rn6.ncbiRefSeq) to identify the genes using the RNA STAR algorithm. Differential expression analysis was performed using DESeq2, which has a built-in normalization function for accurate assessment of changes in gene expression.

A text file of differentially expressed genes (DEGs) from DESeq2 was uploaded to MitoMiner (MitoMiner 4.0: Home (cam.ac.uk)) to locate mitochondria-related genes and generate an interaction network based on functions and known interactions. The resulting list of genes that were altered, the fold change, and the significance (represented as the p-value) of the change were uploaded onto the Search Tool for the Retrieval of Interacting Genes/Proteins (STRING) (STRING-db) analysis [67] to make pathway associations and network interactions.

Following RNA-sequencing, key pathways were identified by Qiagen’s web-based software: Ingenuity Pathway Analysis (IPA) (QIAGEN Inc., https://digitalinsights.qiagen.com/IPA) [68]. Possible pathways were organized by significance (false discovery rate, FDR) and fold change (FC), so the most likely activated pathways (highest FC and FDR) were investigated.

### Ex vivo studies

#### Human explant culture and treatment

Ex vivo retinal explants (*n* = 5 donors: 3 males, 2 females) were obtained within 24 h postmortem human eyes from UNTHSC’s Willed Body Program. Six to eight 4 mm punches were retrieved from each retina and placed RGC side up on Corning Transwell polycarbonate membrane cell culture inserts (Millipore Sigma) in a 24-well plate and kept in culture media (Neurobasal A Medium, B27 supplement, N_2_ supplement, Penicillin/Streptomycin, L-Glutamine) and incubated at 37 °C with 5% CO_2_. Three to four retinal punches were immediately fixed in 4% PFA as a 0-day control (0 days ex vivo; 0 dev) while the remaining punches (3-4 punches per group) were treated with either vehicle (PBS) or 12.5 μg/mL CPP-P1 for 7 days in culture medium, followed by fixation and staining with RGC marker, RBPMS. Cell counting was performed semi-manually using ImageJ software.

#### CREB Inhibition

Approximately 1.5 g of potent and selective CREB inhibitor 666-15 (cat: 5383410001, Millipore Sigma, Burlington, MA) was dissolved in DMSO to a stock concentration of 2.5 mM, which was then further diluted in sterile water for a 10% concentration of DMSO at 250 μM, which was then diluted 1000-fold in the explant culture media to a final concentration of 0.01% DMSO and 250 nM of 666-15. Ex vivo retinal explant punches (*n* = 3 donors) were treated with vehicle or 12.5 μg/mL CPP-P1 and with or without 250 nM of CREB inhibitor 666-15 [69–72].

### Statistical analysis

The statistical analyses were performed using GraphPad Prism 9 (GraphPad Software, La Jolla, CA). A one-way ANOVA followed by Tukey’s multiple comparison test or an unpaired *t*-test was performed. Data are presented as mean ± standard error of the mean (SEM). Normality was assessed with the Shapiro-Wilk test (N of 3-4 biological replicates). *P*-values less than 0.05 were considered statistically different (*p < 0.05, **p < 0.01, ***p < 0.001, ****p < 0.0001).

### Supplementary information

Supplementary figure 1 - original data

Supplemental Figure 2

## Data Availability

The datasets generated and analyzed during the current study are available from the corresponding author upon reasonable request. RNA sequencing data is available through the NCBI database in the Sequence Read Archive (SRA) format under the accession number PRJNA1061184.
