# Supplementary figures and images for: Mechanisms contributing to inhibition of retinal ganglion cell death by cell permeable peptain-1 under glaucomatous stress
Source: Cell Death Discov. 2024 Jun 28;10:305. doi: 10.1038/s41420-024-02070-8 (PMC11213865; doi:10.1038/s41420-024-02070-8)

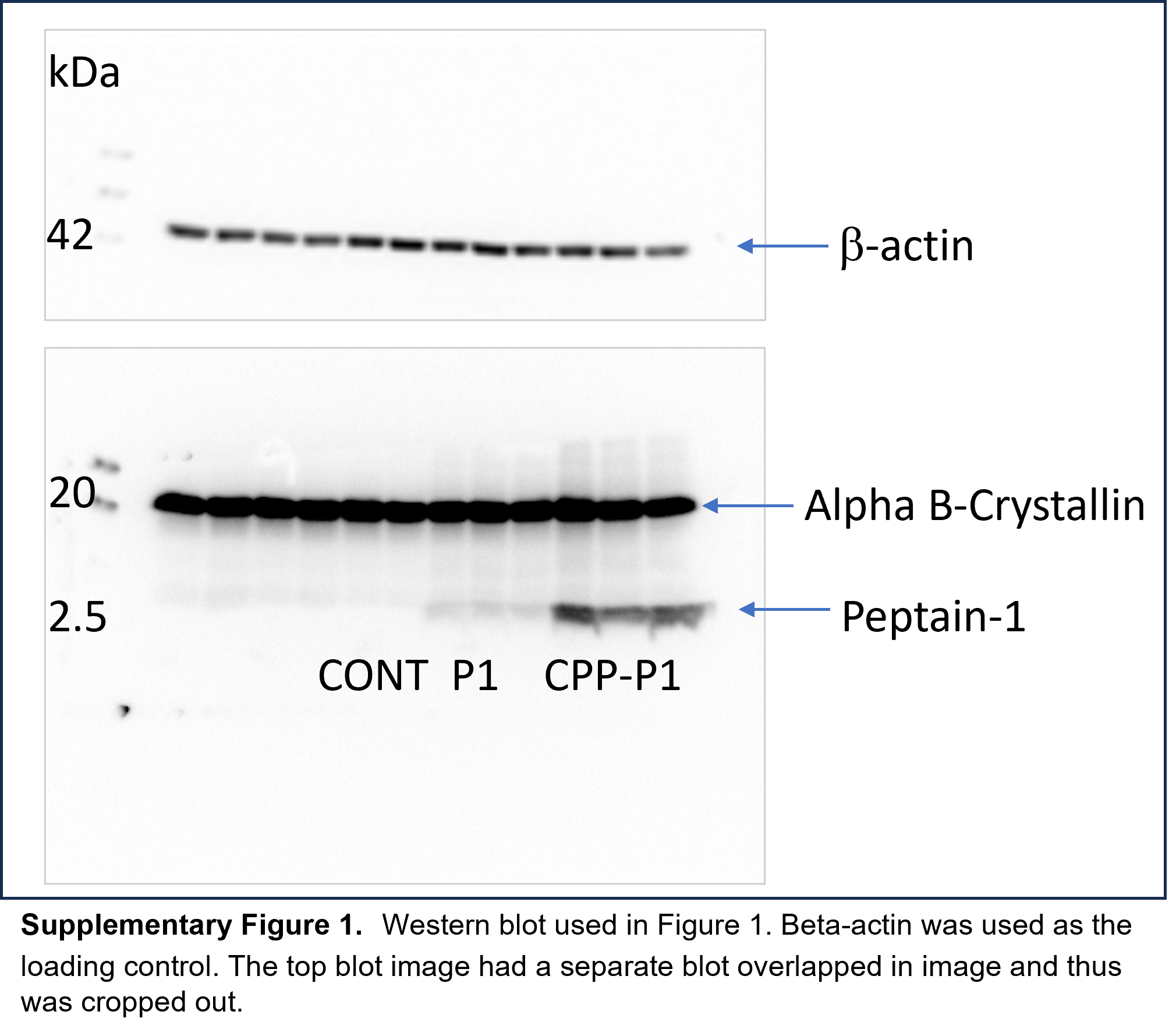

Supplement: Supplementary file 1 — Supplementary figure 1 - original data [file 41420_2024_2070_MOESM1_ESM.png]

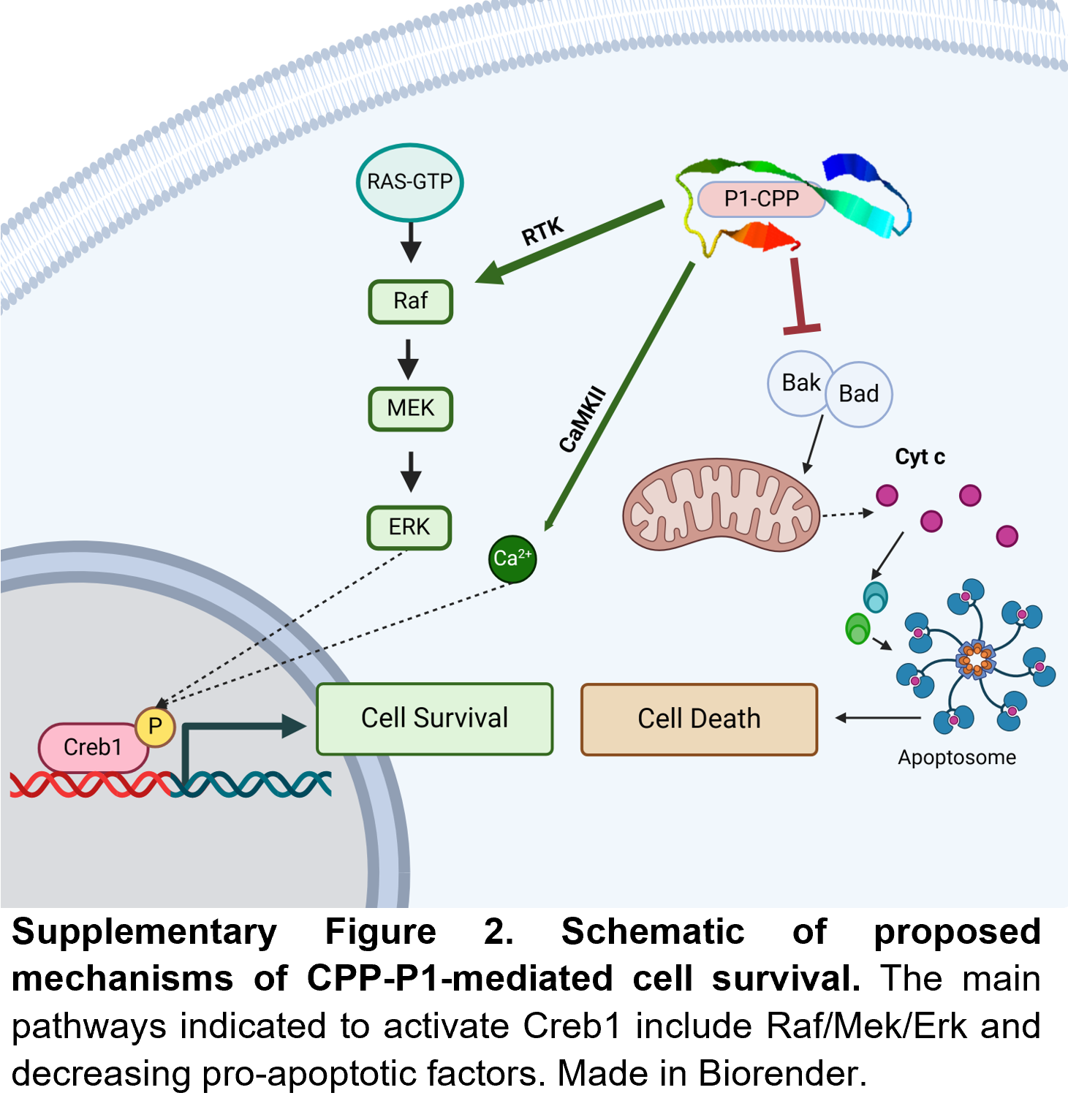

Supplement: Supplementary file 2 — Supplemental Figure 2 [file 41420_2024_2070_MOESM2_ESM.png]
